# The complete chloroplast genome of *Leonurus sibiricus* Linnaeus (Labiatae, Leonurus Miller)

**DOI:** 10.1080/23802359.2024.2383673

**Published:** 2024-07-26

**Authors:** Yan-chang Huang, Wen-xiao Men, Che Bian, He-fei Xue, Wen-juan Hou, Yue-Yue Song, Yan-yun Yang, Liang Xu

**Affiliations:** School of Pharmacy, Liaoning University of Traditional Chinese Medicine, Dalian, China

**Keywords:** Chloroplast genome, phylogenetic tree, *Leonurus sibiricus* Linnaeus, Lamiaceae

## Abstract

*Leonurus sibiricus* Linnaeus 1753, an annual or biennial herb found in northern China, Mongolia, and Russia, typically grows in stony, sandy grasslands, and pine forests. This study sequenced and reported the complete chloroplast genome of *L. sibiricus* for the first time. The entire circular genome measures 151,689 bp in length, with a GC content of 38.4%. A total of 133 genes were annotated, including 88 protein-coding genes, 37 tRNAs, and eight rRNAs. The genome exhibits a typical quadripartite structure, comprising a large single-copy (LSC 82,820 bp) region, a small single-copy (SSC 17,619 bp) region, and a pair of inverted repeat (IR 25,625 bp each) regions. Phylogenetic analysis using the maximum-likelihood method indicates that *L. sibiricus* is most closely related to *L. japonicus* Houttuyn. This study provides valuable genomic resources for further research on the phylogenetics and biodiversity of the genus Leonurus.

## Introduction

*L. sibiricus* is native to Inner Mongolia, northern Hebei, Shanxi, and Shaanxi in north China, belonging to the genus *Leonurus* in the Lamiaceae family (Li et al. 1753). It has medicinal value and is considered an ethnomedicinal plant. In China, *L. sibiricus* is used similarly to *L. japonicus*. The entire herb is medicinal, known in Chinese as Yi Mu Cao, which means ‘good for women’ (Miao et al. 2019). Folk medicine primarily employs it for treating gynecological issues such as menoxenia, dysmenorrhea, and amenorrhea (Shang et al. 2014). Ancient texts like the *Shennong Bencao Jing* and the *Compendium of Materia Medica* highlight its use in treating skin itching and blood disorders. Modern research indicates that both *L. sibiricus* and *L. japonicus* contain leonurine, an alkaloid (Li et al. 2024). *L. sibiricus* is rich in diterpenoids (Narukawa et al. 2014), which have been studied for their effects on airway remodeling, anti-inflammatory activity, and anticancer properties (Wieczfinska et al. 2020; Wang et al. 2023). This study sequenced the chloroplast genome of *L. sibiricus* to support species taxonomy and DNA barcoding of the genus Leonurus.

## Materials and methods

### Plant material

**Figure 1.**
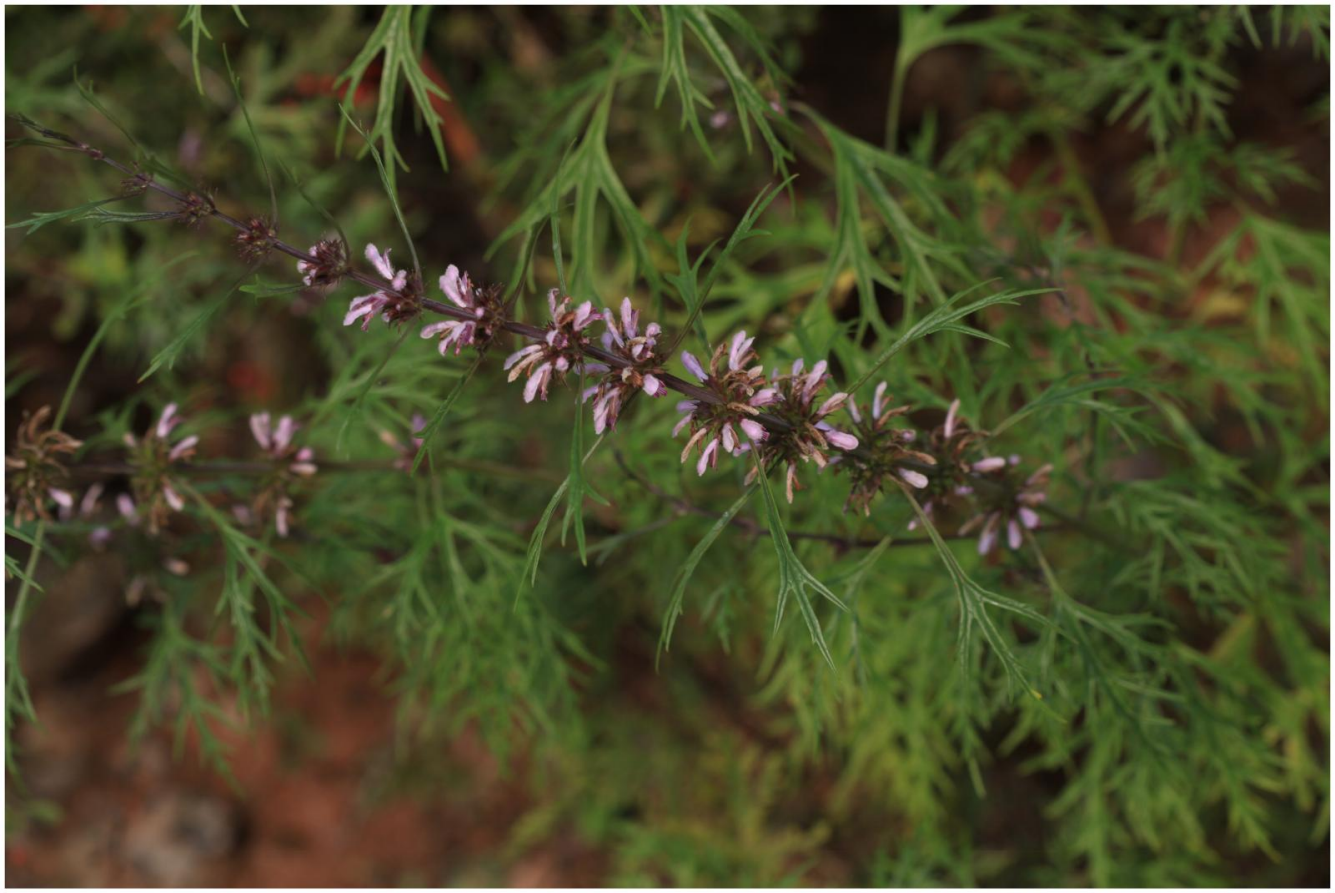
*Leonurus sibiricus* Linnaeus Leaf blade ovate, base broadly cuneate, 3-palmatisect; verticillasters many flowered, Calyx tubular-campanulate. Photograph of *L. sibiricus* taken by Shu-mei Zhang.

Fresh leaves were collected from the Inner Mongolia Autonomous Region, China (N39°49′13.61″, E108°38′22.54″) (Figure 1). The species was identified by Professor Liang Xu of Liaoning University of Traditional Chinese Medicine. The voucher specimen was preserved in the herbarium of the Liaoning University of Traditional Chinese Medicine (Dalian, China) (Liang Xu 861364054@qq.com, *L. sibiricus* number: 210905190808024LY).

### DNA extraction and sequence

Total DNA was extracted from 150 mg of fresh leaves using the cetyltrimethyl ammonium bromide method (Doyle and Doyle 1987). DNA degradation and contamination were monitored on 1% agarose gels. The DNA concentration was measured using the Qubit^®^ DNA Assay Kit in Qubit^®^ 3.0 Flurometer (Invitrogen, Carlsbad, CA). An aliquot of purified DNA (1 μg) was then fragmented to construct a short-insert (350 bp) library using the Nextera XT DNA library preparation kit (Illumina, San Diego, CA). The library was sequenced using the Illumina NovaSeq 6000 platform (San Diego, CA), and the coverage depth was measured using Samtools.

### Genome assembly and annotation

Raw data were processed with NGS QC Toolkit v2.3.3 (https://nipgr.ac.in/ngsqctoolkit.html) (Patel and Jain 2012). High-quality sequence data (4.72 G) were then selected for the *de novo* assembly of the complete chloroplast genome using the assembler SPAdes v. 3.14.1 (http://cab.spbu.ru/software/spades/) (Bankevich et al. 2012). Finally, the complete chloroplast genome was annotated using PGA (Qu et al. 2019) with the chloroplast genome of *Leonurus sibiricus* Linnaeus (NC 067787) as a reference. The annotation of rRNA was done by submitting the sequences to the RNAmmer 1.2 Server (http://www.cbs.dtu.dk/services/RNAmmer/) for prediction, supplemented by homologous sequence alignment and correct boundary range.

### Phylogenetic analysis

Likelihood is a measure proportional to the probability of observing data given the parameters specifying an evolutionary model and branch lengths in the tree (Whelan 2008). To analyze the evolutionary relationship of *L. sibiricus*, 21 complete chloroplast genomes of plants in the Lamiaceae family were selected from NCBI. *Orobanche coerulescens* (Orobanchaceae) is an outer group. MAFFT version 7.037 (Katoh and Standley 2013) was used to identify common protein-coding genes from 23 chloroplast genomes and to compare *L. sibiricus* chloroplast genome with 22 other complete chloroplast genomes using the FFT-NS-2 strategy. The gaps in the alignment have been trimmed with the Gblocks (Version 0.91b, http://molevol.cmima.csic.es). A phylogenetic tree of the 22 chloroplast genomes was constructed using IQ-TREE-1.6.12 (http://www.iqtree.org/) based on the maximum-likelihood method with 1000 bootstrap replications and the JTT + F + R2 model, which was selected using ModelFinder (Kalyaanamoorthy et al. 2017).

## Results

### Genome structure analysis

**Figure 2.**
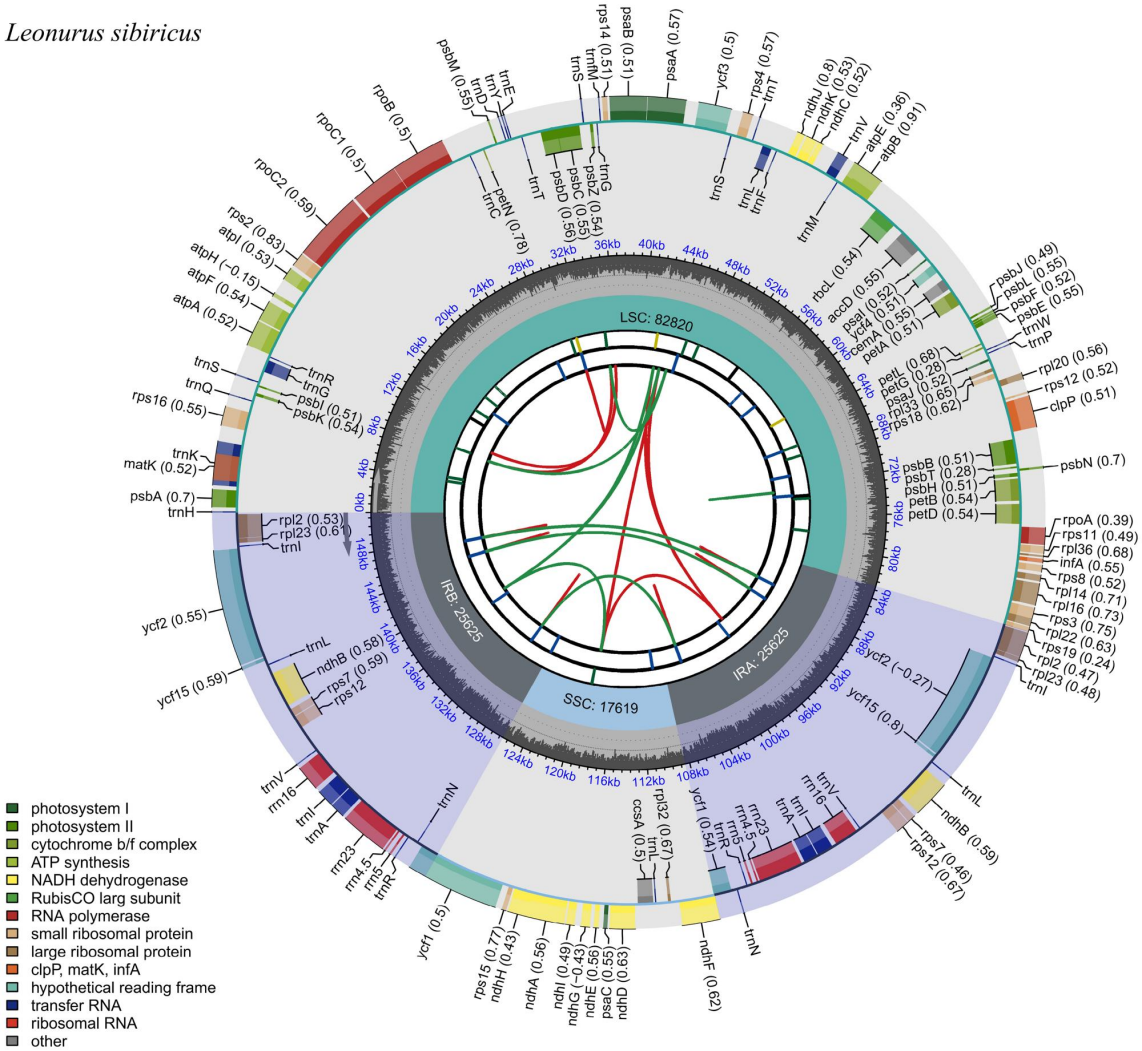
The circular map of the chloroplast genome of *Leonurus sibiricus* Linnaeus has been drawn with the cpgview. From the inside out, the first circle shows the forward and reverse repeats connected with red and green arcs. The second and third circles show the tandem repeats and microsatellite sequences marked with short bars. The outer circle shows the gene structure of the chloroplast genome. Genes have been colored according to their functional categories, shown in the lower left corner.

The length of the sequenced *L. sibiricus* chloroplast genome is 151,689 bp, consisting of four parts: the large single-copy (LSC, 82,820 bp) region, the small single-copy (SSC, 17,619 bp) region, and two inverted repetitive sequences (IRs, 25,625 bp × 2). There are 133 coding genes, including 88 protein-coding genes, eight rRNA genes, and 37 tRNA genes, with a GC content of 38.4%. The *trn*K-UUU, *rps*16, *trn*G-UCC, *atp*F, *rpo*C1, *trn*L-UAA, *trn*V-UAC, *pet*B, *pet*D, *rp*l16, *rpl*2, *ndh*B, *trn*I-GAU, *trn*A-UGC, and *ndh*A genes contain one intron each, the *clp*P and *ycf*3 genes contain two introns and there is a trans-splicing condition in the *rps*12 gene. The chloroplast genome of *L. sibiricus* was correctly assembled based on the coverage depth (average sequencing depth was 2254.83×, maximal sequencing depth was 3784×, minimal sequencing depth was 884x) (Figure S1). The maps of the annotated chloroplast genome, the cis-splicing genes, and the trans-splicing genes of *L. sibiricus* (Figure 2, Supplemental Figures S2 and S3, respectively) were generated using CPGview (Liu et al. 2023).

**Figure 3.**
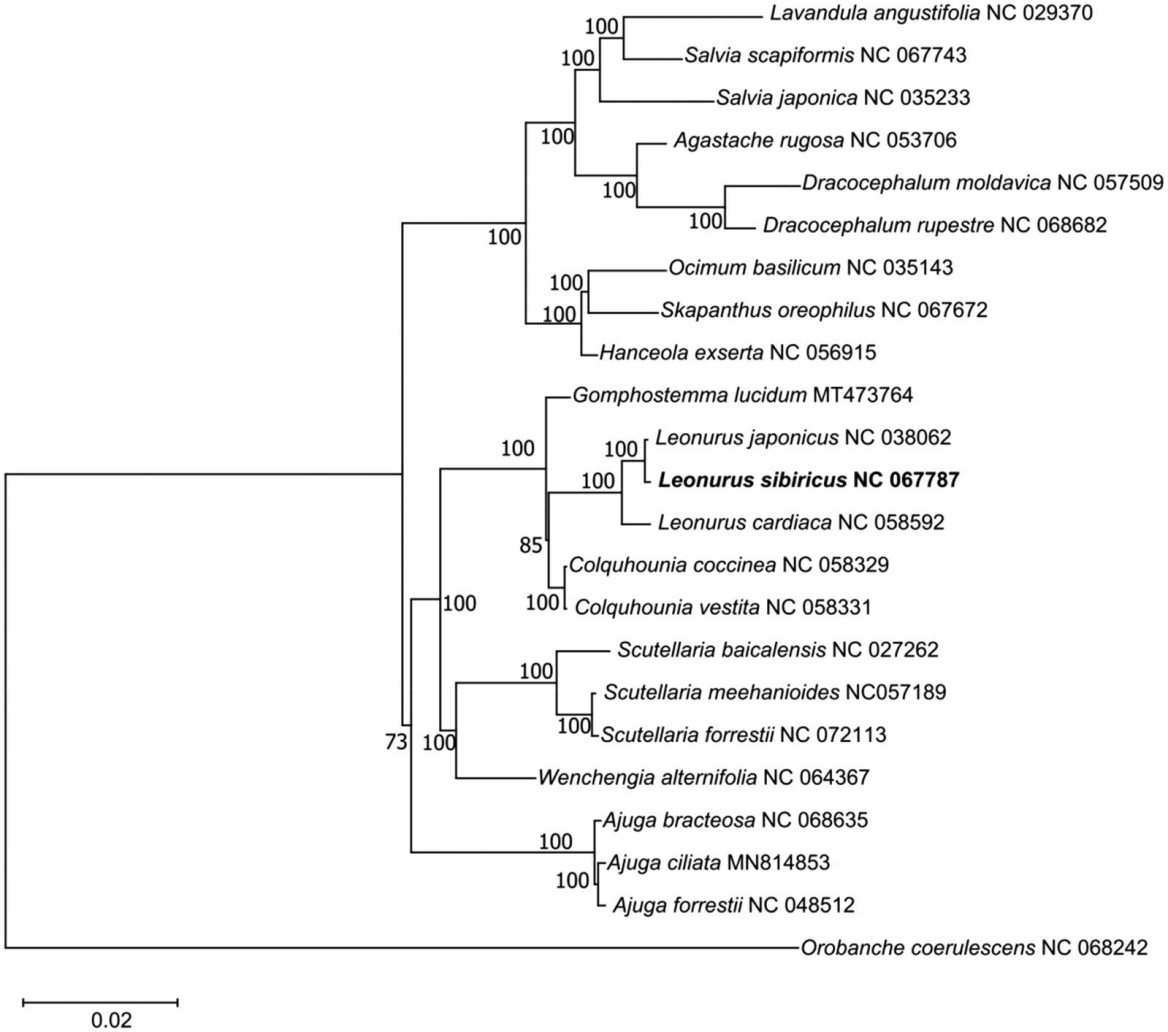
Maximum-likelihood phylogenetic tree of intact chloroplast genomes of Leonurus heterophyllus and 22 other species. The number above the branches represents the bootstrap value for the ML analysis. The optimal evolutionary model was identified as JTT + F + R2 and selected using ModelFinder. The scale bar in the bottom left corner of the figure represents evolutionary distance, with a unit length of 0.02. The following sequences were used: *Lavandula angustifolia* NC029370 (Ma 2018), *Salvia scapiformis* NC067743, *Salvia japonica* NC035233 (Sudarmono and Okada 2007), *Agastache rugosa* NC053706 (Wang et al. 2021), *Dracocephalum moldavica* NC057509 (Yao et al. 2020), *Dracocephalum rupestre* NC068682 (Han et al. 2023), *Ocimum basilicum* NC035143 (Kirankumar et al. 2023), *Skapanthus oreophilus* NC067672, *Hanceola exserta* NC056915 (Zhu et al. 2023), *Gomphostemma lucidum* MT473764, *Leonurus japonicus* NC038062 (Yang et al. 2006), *Leonurus sibiricus* NC 067787, *Leonurus cardiaca* NC058592 (Sun et al. 2021), *Colquhounia coccinea* NC058329, *Colquhounia vestita* NC058331, *Scutellaria baicalensis* NC027262 (Jiang et al. 2023), *Scutellaria meehanioides* NC057189 (Zhang et al. 2021), *Scutellaria forrestii* NC072113, *Wenchengia alternifolia* NC064367, *Ajuga bracteosa* NC068635 (Shang et al. 2023), *Ajuga ciliata* MN814853, *Ajuga forrestii* NC048512 (Tao et al. 2019), and *Orobanche coerulescens* NC068242.

### Genome structure analysis

The phylogenetic tree indicates that, in comparison to the other species, the genetic distance between this species and the outgroup is relatively long (Figure 3). *L. japonicus*, *L. sibiricus*, and *Leonurus cardiaca* are on the same branch, with *L. japonicus* and *L. sibiricus* having the closest evolutionary relationship.

## Conclusions and discussion

This study presents the first complete chloroplast genome of *L. sibiricus*, measuring 151,689 bp with a quadripartite structure. The phylogenetic tree reveals a close relationship between *L. sibiricus* and *L. japonicus*.

These findings support morphological classification and provide data for evolutionary studies, species identification, and resource development within the Lamiaceae family.

## Supplementary Material

Supporting materials.docx

## Data Availability

The genome sequence data supporting the results of this study are openly available in GenBank at NCBI (https://www.ncbi.nlm.nih.gov/) under accession number NC067787. The associated BioProject, SRA, and Bio-Sample numbers are PRJNA874872, SRR21312611 (Illumina), and SAMN30578237, respectively.
