# Supplementary material for: The complete chloroplast genome of Leonurus sibiricus Linnaeus (Labiatae, Leonurus Miller)
Source: Mitochondrial DNA B Resour. 2024 Jul 26;9(7):934–8. doi: 10.1080/23802359.2024.2383673 (PMC11285220; doi:10.1080/23802359.2024.2383673)
Supplement: Supporting materials.docx [file TMDN_A_2383673_SM4500.docx]

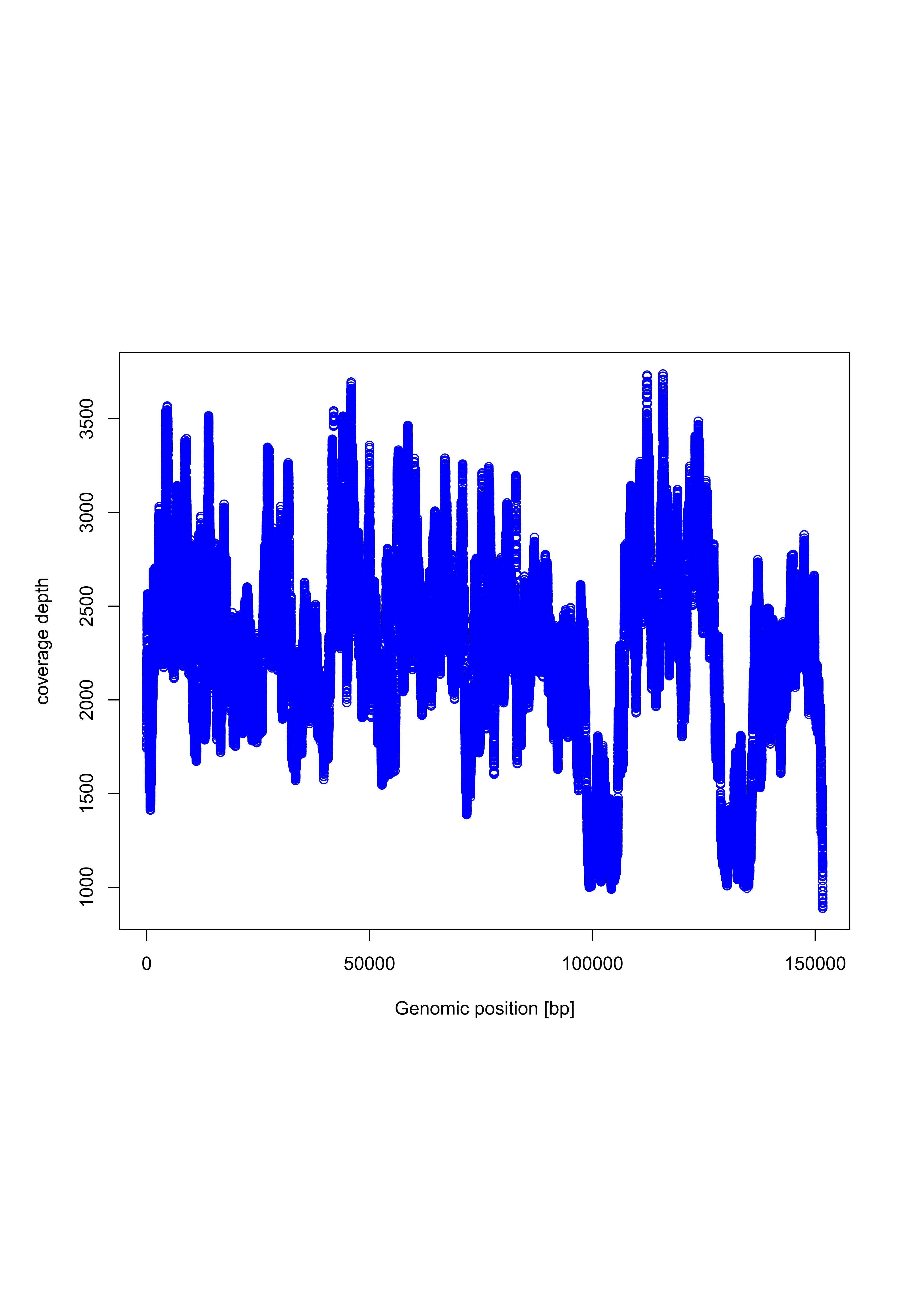


**Figure S1.** Sequencing coverage depth of *Leonurus sibiricus* Linnaeus**.** The illumina 4 short sequences were compared to the chloroplast genome sequences using BWA software and finally the coverage was calculated using samtools depth (High coverage of over 100X). The horizontal coordinate is the chloroplast length and the vertical coordinate is the coverage depth.

LiH. 2013. Aligning sequence reads, clone sequences and assembly contigs with BWA-MEM. arXiv Prepr arXiv. 0(0):3.

LiH. et al. 2009 The Sequence Alignment/Map format and SAMtools. Bioinformatics,25, 2078-2079.


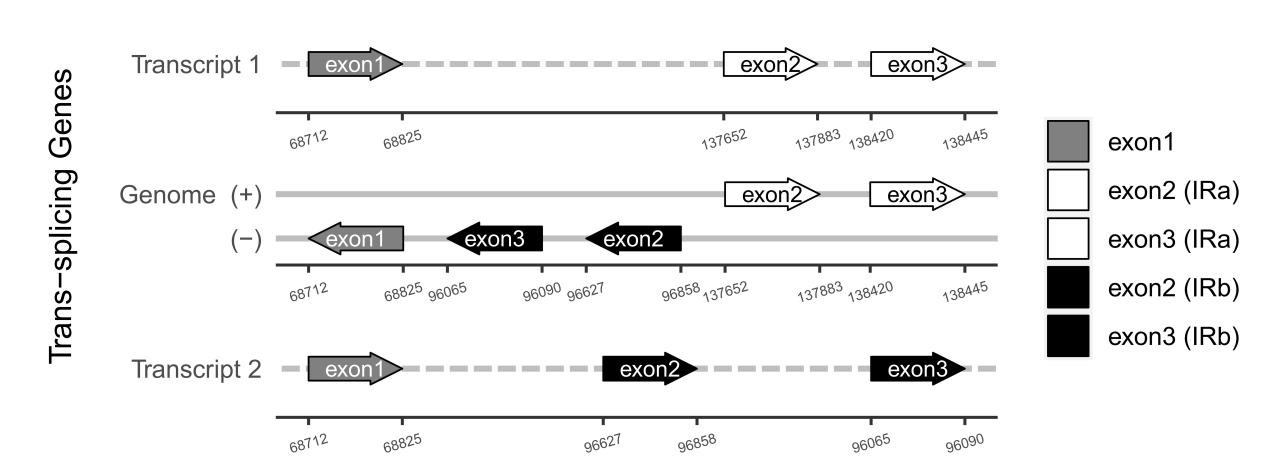


**Figure S2.** Schematic map of the trans-splicing gene rps12 in the chloroplast genome.

It has three unique exons. Two of them are duplicated as they are located in the IR regions.





**Figure S3.** Schematic map of the cis-splicing genes in the chloroplast genome. The genes are arranged from top to bottom based on their order on the chloroplast genome.

The gene names are shown on the left, and the gene structures are on the right. The exons are shown in black; the introns are shown in white. The arrow indicates the sense direction of the gene. Please note that lengths of exons and introns are not drawn to scale.
